# Surface Topography of Hardened Stainless Steel in Dry Finish Turning Using CBN and Cemented Carbide Inserts

**DOI:** 10.3390/ma19061103

**Published:** 2026-03-12

**Authors:** Kamil Leksycki, Eugene Feldshtein, Jakub Pawłowski

**Affiliations:** Institute of Mechanical Engineering, University of Zielona Gora, 4 Prof. Z. Szafrana Street, 65-516 Zielona Gora, Poland; j99pawlowski@gmail.com

**Keywords:** hardened X20Cr13 stainless steel, dry finish turning, turning parameters, surface topography, CBN and cemented carbide (CC) inserts

## Abstract

The proper selection of surface topography (ST) parameters is crucial for ensuring the effective performance of machine components, including their wear and corrosion resistance. In the literature, research on the ST of hardened stainless steels (SSs) after finish turning using cubic boron nitride (CBN) inserts, as well as comparisons with cemented carbide (CC) inserts depending on cutting parameters, is still limited. In this study, the ST of X20Cr13 martensitic hardened SS under dry finish turning with various cutting speeds and feed rates was investigated. Experiments were conducted using a CNC lathe with CBN and CC inserts. A Sensofar S Neox 3D optical profilometer was employed to characterize the ST features, including height surface roughness (SR) parameters, SR profiles, and 2D and 3D surface images. The Parameter Space Investigation method was used to design the experimental plan. For both CBN and CC inserts, the feed rate was the dominant factor influencing the overall SR, described by the Sa and Sq parameters. The extreme parameters Sp, Sv, and Sz were determined by the relationship between feed rate and cutting speed. With appropriately selected turning parameters, it is possible to obtain low Sa values (0.4–0.6 µm), which can eliminate the need for grinding operations. CBN inserts ensured a more regular shape of the ST, while CC inserts contributed to a wavy surface characteristic, associated with more intense plastic deformation. However, low Sa values may be accompanied by isolated peaks, indicating that this parameter does not always fully reflect the presence of extreme micro-irregularities. On the machined surfaces, adhesive bonds of chips and cutting tool material were observed. In addition, micro-scratches were registered for CBN inserts, and a side flow phenomenon for CC inserts. The results confirm that dry turning of hardened SSs can be effectively performed using both CC and CBN inserts.

## 1. Introduction

The development of modern manufacturing technologies imposes increasingly stringent requirements on structural materials and their processing methods. Due to their excellent operational properties, including resistance to aggressive environments and high strength, stainless steels (SSs) are widely used in the chemical, energy, food, medical, aerospace, and automotive industries, among others.

Several studies have described the use of state-of-the-art tool materials in the machining of structural materials. Tu et al. [1] examined the adhesion and diffusion mechanisms of cubic boron nitride (CBN) tools during high-speed cutting of Ni-based alloys. The thickness of the CBN adhesion layers was about 20–60 μm, while the diffusion transition layer between CBN and the alloy measured approximately 28–65 nm. A number of Cr-, Ni-, and B-based compounds were also observed. Quantitative analysis indicated that the dislocation density in CBN grains was approximately 7.0 × 10^15^/m. Bjerke et al. [2] investigated the wear mechanisms of polycrystalline cubic boron nitride (PCBN) inserts, both uncoated and coated, during the turning of 17–4 PH SS at speeds of 200–600 m/min. Adhesive wear was active only at low speeds, whereas increasing the cutting speed led to higher wear due to diffusion and oxidation. Oxidation accelerated PCBN wear and promoted the formation of metal oxides in the build-up edge (BuE). Boing et al. [3] studied the wear mechanisms of two types of polycrystalline diamond (PCD) tools when machining Al-based MMCs. The PCD grade with larger grain size produced a rougher topography of the worn surface. Analysis of the cutting inserts revealed two types of wear: micro-wear and macro-wear, both caused by detachment of diamond grains from the PCD matrix. Tian et al. [4] examined the cutting characteristics of PCBN tools, including tool wear and tool life, cutting forces, cutting temperature, and surface roughness (SR) during the machining of Fe-based superalloys. Wear of the rake surface was primarily determined by adhesion, chipping, flaking, and groove formation. At relatively higher cutting speeds, BuE and micro-cracks also appeared. Zhang et al. [5] analyzed conventional turning (CT) and ultrasonic vibration turning (UVT) of Ni-based superalloys using a single-crystal diamond (SCD) tool. SR, morphology, chip formation, tool wear, and subsurface damage were examined. Compared to CT, UVT reduced SR (*Sa* parameter) from 60 nm to 4.815 nm. During CT, significant grain refinement and plastic deformation near the subsurface were observed, primarily due to high thermo-mechanical stresses caused by tool wear. Under CT conditions, the work-piece material underwent substantial hardening. In contrast, subsurface damage thickness decreased from 2.27 μm under CT to 0.1902 μm under UVT. Xu et al. [6] analyzed diamond tool wear mechanisms when machining iron-based materials, including the influence of diamond crystallographic plane orientation and strategies to reduce tool wear. Wang et al. [7] used CBN tools for turning GX23CrMoV12-1 steel, investigating wear curves, friction behavior, wear and fracture mechanisms, and machined surface morphology. The dry friction coefficient of CBN was approximately 0.2. He et al. [8] studied the wear resistance and performance of CBN tools under various cutting conditions for Vit1 metallic glass. Cutting parameters influenced the stages of tool wear, which included abrasive, diffusion, oxidative, and adhesive mechanisms. SR increased with tool wear and chip adhesion. Thamizhmanii et al. [9] examined wear on the clearance surface of CBN tools when turning titanium and Inconel 718 alloys. Surface quality depended on tool wear and increased downtime, necessitating frequent tool changes. Liao et al. [10] evaluated machinability parameters to assess overall machining performance, considering tool wear, cutting forces and temperature, chip shape and brittleness, and surface integrity. Tool life depended on cutting speed, with an optimum of 200 m/min. CBN tool wear was characterized by brittle fracture and adhesive wear. At 200–300 m/min, the frequency of CBN particle breakage decreased, and no cracking occurred, although abrasive wear increased. The *Sa* parameter initially decreased but increased with tool wear. Khetre et al. [11] demonstrated the effectiveness of CBN tools when machining Inconel alloys under MQL conditions, using coconut oil with silicon carbide (SiC) and multi-layer carbon nanotube additives. Tuan et al. [12] tested the effect of MQL parameters using MoS_2_ nano-oil on SR and cutting forces during hard turning of 90CrSi hardened steel with CBN inserts. Özdemir et al. [13] analyzed the influence of cutting conditions on cutting forces and SR during dry hard turning of AISI 4140 steel, using coated carbide and CBN inserts with varying nose radii. Zębala et al. [14] studied longitudinal turning of sintered nickel-cobalt alloy with CBN tools, focusing on cutting forces and specific cutting forces considering cutting edge wear. Latosińska et al. [15] investigated longitudinal turning of Inconel 718 alloy parts produced by Direct Metal Laser Sintering using CBN inserts, examining the effects of cutting conditions on cutting forces, SR, and chip shape. Zhou et al. [16] evaluated high-speed turning of AD730^®^ and IN718 superalloys with CBN inserts. Surface defects, including carbide cracks, plastic deformation, and smearing, were observed, affecting SR. Significant plastic deformation was detected in subsurface layers. Lin et al. [17] described material removal mechanisms and micro-structural evolution of tungsten alloys under vibration turning with PCD and SCD tools. PCD tools exhibited gradual wear through grain detachment, whereas SCD tools failed abruptly via edge chipping. SR parameters were comparable (PCD—*Sa* ~65 nm, SCD—*Sa* ~26 nm), but the mechanisms of surface morphology and subsurface micro-structure evolution differed significantly.

In summary, it can be stated that, in recent years, information on the efficiency of machining modern structural materials has been increasingly reported, although the results remain inconsistent. Among the main issues are: (i) the lack of detailed data on the surface topography (ST) of hardened SSs after finish turning with super-hard materials; (ii) in studies on CBN materials, researchers focus primarily on practical aspects (chips, cutting forces, tool wear), whereas in the case of PCD materials, investigations typically concentrate on an in-depth analysis of the machined surface morphology and submicro-structure; (iii) comparisons of three-dimensional STs depending on cutting parameters are usually absent when considering the details of surface layer formation.

Stainless steels are commonly classified into several groups, including austenitic, ferritic, duplex, precipitation-hardened, and martensitic grades. Austenitic and ferritic stainless steels typically exhibit high corrosion resistance but moderate mechanical strength, whereas duplex steels combine ferritic and austenitic phases. In contrast, martensitic stainless steels can attain high hardness and strength after heat treatment, making them suitable for applications requiring enhanced wear resistance. Accordingly, martensitic stainless steel was selected as the material investigated in this study.

The aim of this paper is to present the potential for effective use of PCBN (hereafter referred to as CBN) inserts in dry finish turning of hardened X20Cr13 SS, by analyzing changes in ST depending on cutting parameters, as well as in comparison to the use of cemented carbide (CC) inserts with a PVD coating.

## 2. Materials and Methods

### 2.1. Material

Hardened X20Cr13 (1.4021, AISI 420) SS with medium corrosion resistance and high mechanical strength was used. The chemical composition of X20Cr13 steel (wt%), according to DIN EN 10088-3 [18].

The tested steel is intended for heat treatment and exhibits high mechanical properties (strength and ductility) while maintaining sufficient corrosion resistance. It can be used in environments containing steam, low concentrations of inorganic acids, thinners, pure water, and similar media. The steel can also be classified as heat-resistant up to 825 °C, maintaining stable and sufficiently high mechanical properties within the operating temperature range of 475–500 °C. The steel was quenched and tempered under QT800 conditions. Basic mechanical properties of the heat-treated steel are as follows (according to EN ISO 6892-1 [19] and EN ISO 6506-1 [20]): tensile strength *Rm* 700–850 MPa; yield strength *Rp* 0.2 > 500 MPa; elongation *A* > 13%; impact strength *KV* > 25 J; hardness 255 HB.

This steel grade is used for the production of screws operating at elevated temperatures, turbine blades, food and household knives, surgical instruments, bushings, valve seats, scrapers, blades, shafts, glands, springs, machine components, pressure die-casting molds, and pins subjected to low loads in the automotive, petrochemical, industrial hydraulics, and energy industries.

### 2.2. Turning Conditions

A CLX 350 lathe center (DMG MORI, Pleszew, Poland) was used for the tests with two types of cutting tools.

In the first case, CC inserts with a PVD coating, designated DCMX 11T304-WM GC1115 (Sandvik Coromant, Sandviken, Sweden), and an SDJCL 2020 K11 holder (AKKO, Konya, Turkey) were employed. The double-layer coating (TiAlN + TiAlN) enhances wear resistance at elevated temperatures. In the second case, CBN inserts, designated SNGA 120408S01030A 7025 (Sandvik Coromant, Sandviken, Sweden), and a PSSNL 2020 K12C holder (AKKO, Konya, Türkiye) were used. The insert was designed for turning hard materials, containing 60% CBN with a bimodal grain distribution of 1–3 μm in a Ti(C,N) and Al composite-based binder.

Since hard materials are typically machined with CBN tools without coolant in practice, the turning experiments were conducted under dry machining conditions.

The influence of tool wear on the obtained results was minimized by replacing the cutting insert with a new one before each test.

### 2.3. Measuring Apparatus

The industry increasingly demands measurement methods that ensure high repeatability, rapid acquisition, reliable results, and accurate assessment of machined surfaces. Therefore, the Sensofar S Neox 3D optical microscope (Sensofar Group, Barcelona, Spain) equipped with Mountains Maps Premium 7.4 software (Digital Surf, Besançon, France) was used to measure ST. This device is more efficient and functional than other optical profilers, enabling data acquisition at rates of up to 180 frames per second. The 3D height parameters of ST were analyzed in accordance with ISO 25178-2 [21], namely *Sa, Sz, Sp, Sv,* and *Sq*. The *Sa* and *Sq* parameters describe the general level and the dispersion of surface heights, whereas the extreme parameters *Sp* (maximum peak height) and *Sv* (maximum pit depth) represent the highest peak and the deepest valley relative to the mean plane, respectively. The *Sz* parameter, being the sum of *Sp* and *Sv*, characterizes the total height of the ST. The system also provides 2D and 3D analysis of machined surfaces, including roughness profiles.

### 2.4. Design of Experiments

The Parameter Space Investigation (PSI) method was used to plan the experimental tests, ensuring a minimum number of test points. A detailed description and the conditions for determining the test points were provided by Leksycki and Feldshtein [22].

The coordinates of the test points (Table 1) were calculated within the accepted ranges of cutting speed *v_c_* (100–300 m/min) and feed rate *f* (0.05–0.25 mm/rev). A constant cutting depth of 0.2 mm was applied. The parameter ranges were determined based on data provided by the cutting tool manufacturers.

Statistical analysis of the test results was performed using Statistica 13 software (TIBCO Software Inc., Palo Alto, CA, USA). To control measurement errors, three measurements were taken at each test point.

### 2.5. Experimental Setup

**Figure 1:**
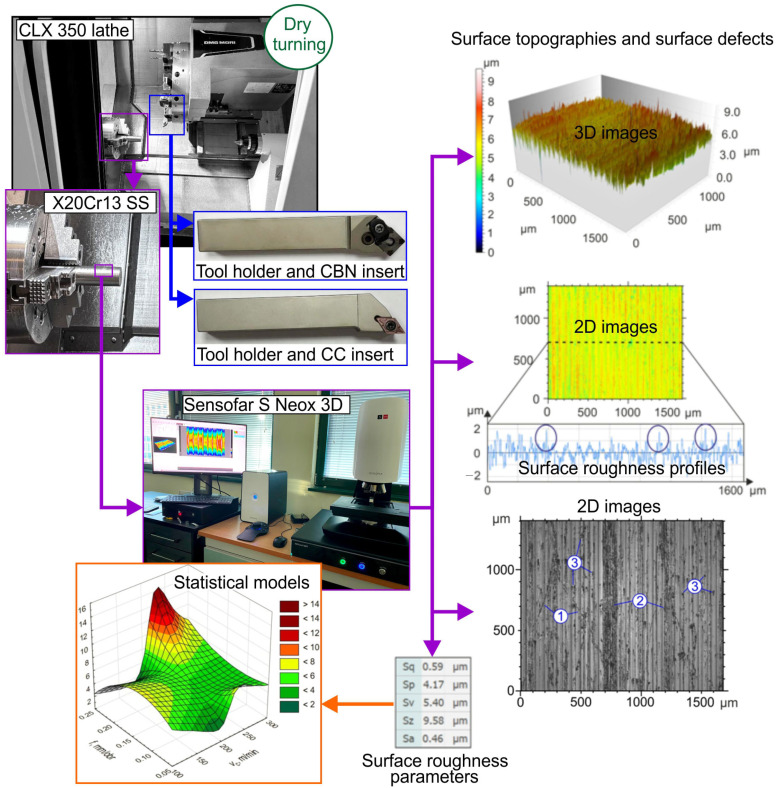
Schematic of the experimental setup.

The schematic diagram of the experimental setup is shown in Figure 1. The diagram presents the applied research and measurement equipment, as well as examples of the obtained experimental results.

## 3. Results and Discussion

The assessment of the quality of machined surfaces is crucial in machining processes, as it directly affects the performance, durability, and reliability of manufactured components [23]. ST parameters determine, among other factors, wear resistance, material fatigue, friction, joint tightness, and susceptibility to corrosion. In many engineering applications, the quality of the surface layer determines whether functional requirements are met, which are often more important than dimensional accuracy [24].

In modern industrial production, particularly with regard to difficult-to-cut materials such as nickel- and cobalt-based alloys, titanium alloys, and SSs, reliable surface quality assessment is an important element of technological process control and of ensuring the repeatability of manufactured products [25,26].

**Figure 2:**
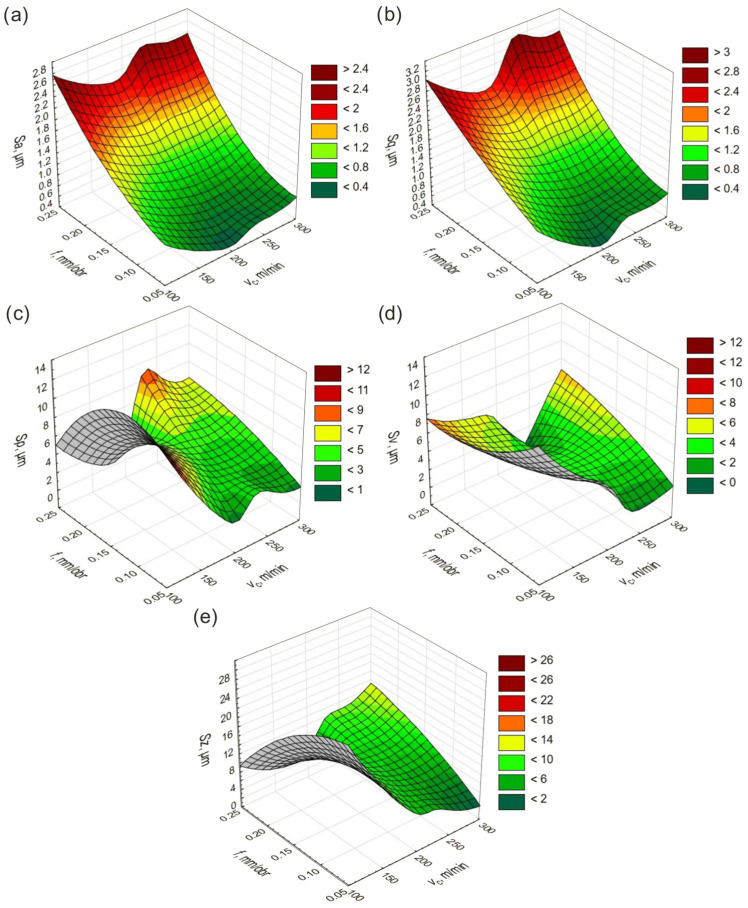
Changes in height parameters as a function of *v*_c_ and *f* after turning with CBN inserts: (**a**) *Sa*, (**b**) *Sq*, (**c**) *Sp*, (**d**) *Sv*, (**e**) *Sz*.

Statistical models of the height SR parameters obtained during turning with CBN inserts are shown in Figure 2.

When using CBN inserts, lower *Sa* and *Sq* values were obtained in the *f* range of 0.05–0.15 mm/rev across the tested range of *v*_c_, whereas higher values occurred in the range of 0.2–0.25 mm/rev over the same *v*_c_ range. Thus, it can be clearly concluded that the overall level and dispersion of surface height described by *Sa* and *Sq* are primarily influenced by the *f*. Lower values of the *Sp, Sv,* and *Sz* parameters were recorded in the range of 0.05–0.1 mm/rev and 200–300 m/min. Additionally, reduced values of *Sp* and *Sz* were observed in the range of 0.2–0.25 mm/rev and 100–200 m/min, whereas lower *Sv* values occurred in the range of 0.2–0.25 mm/rev and 200–250 m/min. Higher *Sp* values were recorded in the ranges of 0.05–0.2 mm/rev and 100–150 m/min, as well as 0.2–0.25 mm/rev and 200–250 m/min, while increased *Sv* and *Sz* values were observed in the range of 0.05–0.15 mm/rev and 100–150 m/min. It can therefore be concluded that the extreme surface height parameters depend on the interaction between *f* and *v*_c_.

**Figure 3:**
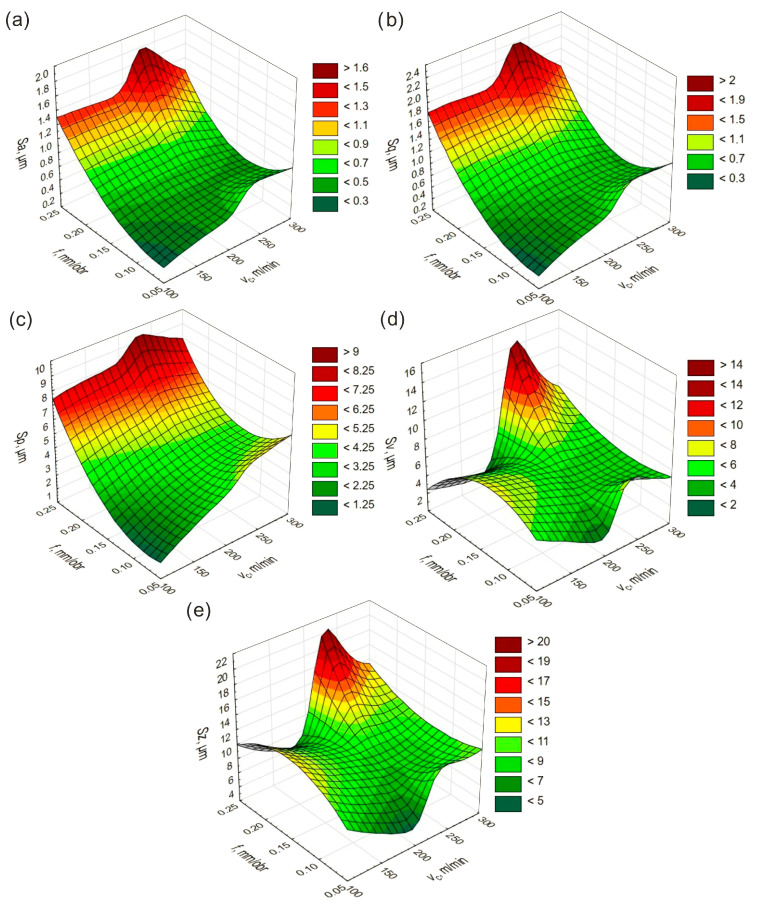
Changes in height parameters as a function of *v*_c_ and *f* after turning with CC inserts: (**a**) *Sa*, (**b**) *Sq*, (**c**) *Sp*, (**d**) *Sv*, (**e**) *Sz*.

Statistical models of the height SR parameters obtained during turning with CC inserts are shown in Figure 3.

When using CC inserts, lower values of *Sa, Sq,* and *Sp* were observed in the *f* range of 0.05–0.15 mm/rev and at *v*_c_ of 100–150 m/min, whereas higher values occurred in the range of 0.2–0.25 mm/rev across the tested range of *v*_c_. Lower values of the *Sv* and *Sz* parameters were recorded in the ranges of 0.05–0.1 mm/rev and 175–225 m/min, as well as 0.2–0.25 mm/rev and 100–175 m/min, while higher values were obtained in the range of 0.2–0.25 mm/rev and 200–300 m/min. The trends describing the influence of *f* and *v*_c_ on the analyzed parameters are similar to those observed during turning with CBN inserts.

According to ISO 286-1 [27], finish turning makes it possible to achieve part tolerances at the IT6–IT7 level. In technological practice, achieving such dimensional accuracy is correlated with obtaining low SR. In particular, achieving *Ra* and *Sa* values below 0.8 µm is characteristic of processes performed with IT7 accuracy, typical of finish turning using tools with high-quality cutting edges, such as CBN tools. This level of ST is comparable to the effects of grinding and, in many applications, eliminates the need for additional finishing operations. For this reason, surfaces machined with *Sa* parameters below 0.8 µm were presented and analyzed in this study.

**Figure 4:**
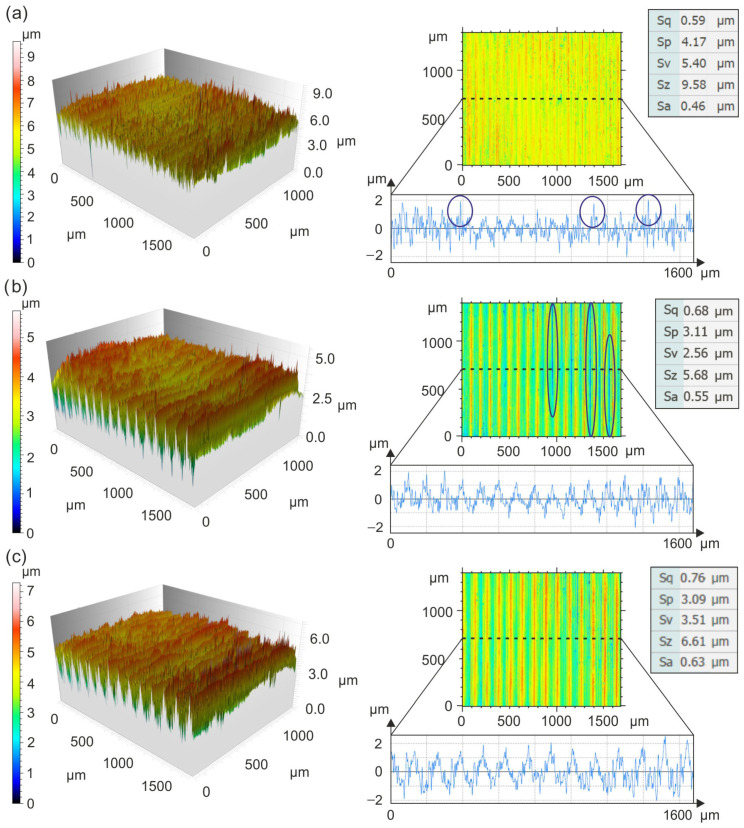
Details of the topography of surfaces machined with CBN inserts: (**a**) 0.075 mm/rev and 175 m/min, (**b**) 0.1 mm/rev and 250 m/min, and (**c**) 0.125 mm/rev and 225 m/min.

Topographical details of surfaces machined with CBN inserts are shown in Figure 4.

The use of CBN inserts for turning at 0.075 mm/rev and 175 m/min ensures *Sa* = 0.46 µm. Despite this low *Sa* value, individual peaks with heights of up to 9 µm can be observed on the machined surface. Turning at 0.1 mm/rev and 250 m/min results in *Sa* = 0.55 µm, with the surface characterized by a regular arrangement of micro-irregularity peaks and varied valley depths. This distribution of micro-irregularities is typical for the turning process and results from its geometric and kinematic conditions [28]. A similar surface shape is observed at 0.125 mm/rev and 225 m/min, with *Sa* = 0.63 µm. Grzesik et al. [29] showed that when turning hardened AISI 5140 steel with ceramic Wiper inserts, the surfaces exhibit flattened elevations and smaller slopes compared to those machined with conventional inserts. Gaitonde et al. [30], Özel et al. [31], and Davim and Figueira [32] compared conventional inserts with ceramic Wiper inserts, showing that turning hardened AISI D2 steel with Wiper inserts provides *Ra* values < 0.5 µm, whereas conventional inserts yield *Ra* values < 0.8 µm. In addition, Chou et al. [33] demonstrated that turning hardened tool steel with CBN can achieve *Ra* values of approximately 0.1 µm.

**Figure 5:**
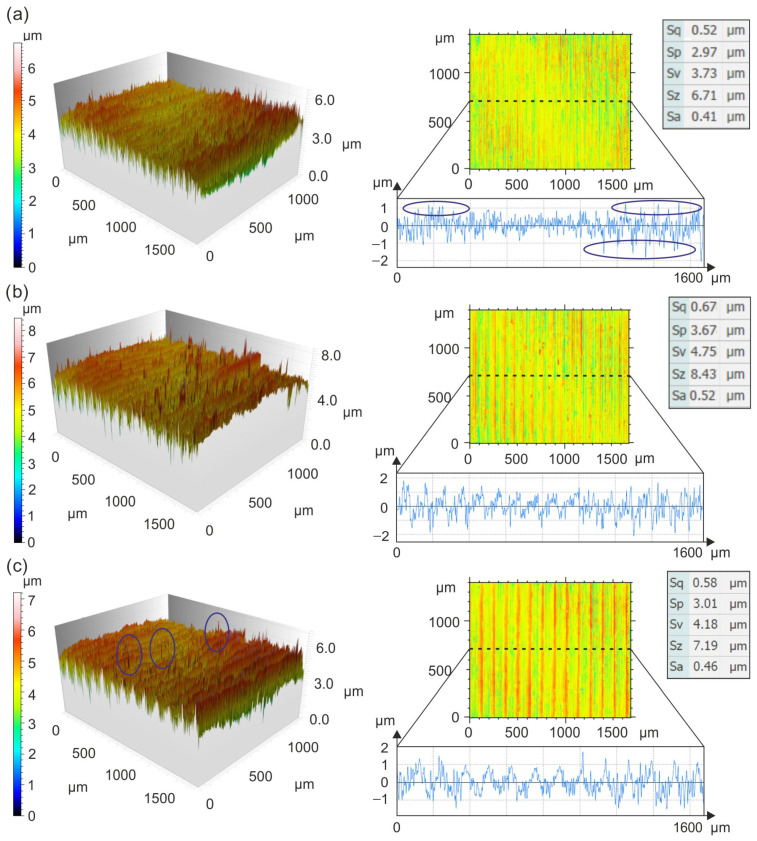
Details of the topography of surfaces machined with CC inserts: (**a**) 0.075 mm/rev and 175 m/min, (**b**) 0.1 mm/rev and 250 m/min, and (**c**) 0.125 mm/rev and 225 m/min.

Topographical details of surfaces machined with CC inserts are shown in Figure 5.

The use of CC inserts for turning at 0.075 mm/rev and 175 m/min ensures *Sa* = 0.41 µm. These machining conditions promote a wavy ST, indicating plastic deformation and resulting from the intense mechanical and thermal impact of the cutting edge on the machined material. This behavior is characteristic of dry machining of ductile materials [34]. Turning at 0.1 mm/rev and 250 m/min results in *Sa* = 0.52 µm. Despite this low *Sa* value, peaks reaching heights of up to 8 µm are observed across the entire machined surface. When turning at 0.125 mm/rev and 225 m/min, *Sa* = 0.46 µm was measured. The surface exhibits a regular distribution of peaks and valleys typical of the turning process, with single peaks also present.

**Figure 6:**
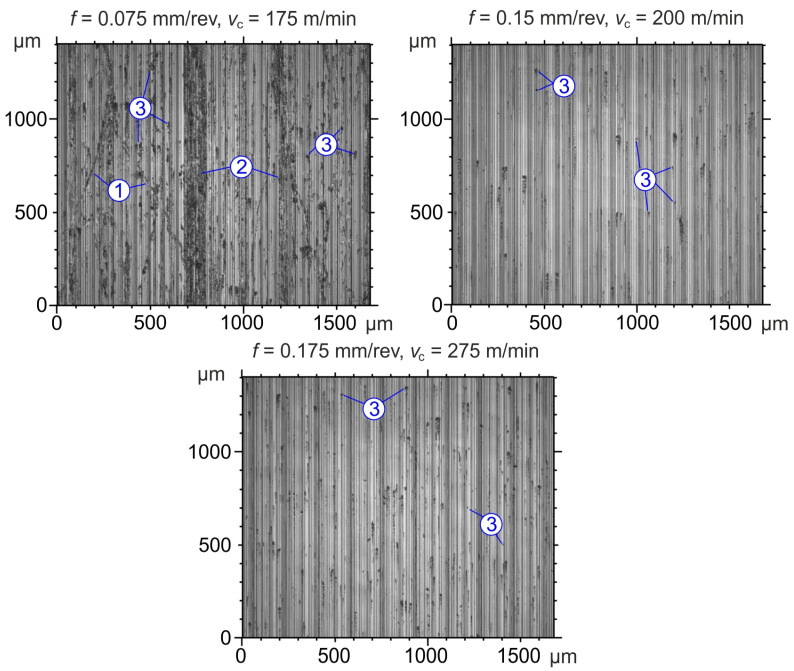
Surface defects after turning with CBN inserts: 1—micro-scratches; 2—adhesive bonds; 3—sticking of fine chips.

2D images of the surfaces machined with CBN inserts are shown in Figure 6.

On the surface machined with CBN inserts at 0.075 mm/rev and 175 m/min, micro-scratches caused by the friction of chips against the machined surface can be observed, along with individual adhesive bonds of cutting tool material and the sticking of fine chips. On surfaces machined at 0.15 mm/rev and 200 m/min, as well as at 0.175 mm/rev and 275 m/min, individual adhesions of fine chips are also observed, with their intensity depending on the interaction between *v*_c_ and *f*.

**Figure 7:**
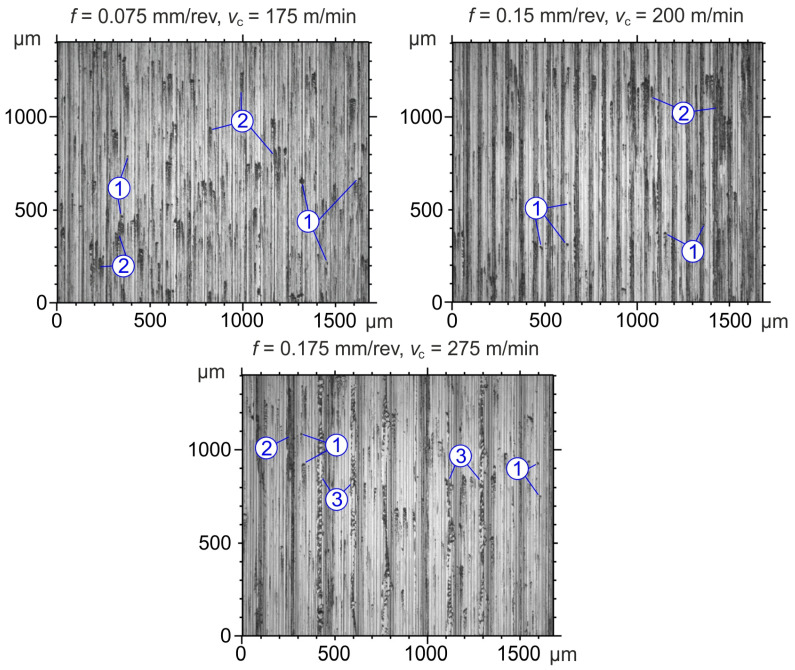
Surface defects after turning with CC inserts: 1—sticking of fine chips; 2—adhesive bonds; 3—side flow phenomenon.

2D images of the surfaces machined with CC inserts are shown in Figure 7.

On the surfaces machined with CC inserts, individual adhesions of fine chips and cutting tool material are observed, similar to surfaces machined with CBN inserts. Additionally, on the surface machined at 0.175 mm/rev and 275 m/min, a side flow phenomenon is observed, which was described in detail by Leksycki [35]. Recent studies by Zhoung et al. [36] indicate that the intensity of side flow increases under the influence of vibrations, and the occurrence of this phenomenon deteriorates surface quality and may cause micro-cracks in the surface layer.

## 4. Conclusions

This paper presents the possibilities for the effective use of CBN inserts in dry finish turning of hardened X20Cr13 SS, through the analysis of ST changes depending on cutting parameters, as well as in comparison with the use of CC inserts with a PVD coating. Based on the conducted study, the following conclusions can be drawn:

- For both CBN and CC inserts, feed rate is the main factor affecting the overall SR, described by the *Sa* and *Sq* parameters. Lower values of these parameters were obtained at smaller feeds (0.05–0.15 mm/rev), while higher values were observed at larger feeds (0.2–0.25 mm/rev), regardless of the tested cutting speed.
- The *Sp, Sv,* and *Sz* parameters are influenced by the interaction between feed rate and cutting speed, with their minimum and maximum values occurring at defined combinations of these turning parameters. The effects of feed rate and cutting speed are similar for both types of inserts, suggesting a comparable mechanism for shaping ST, despite differences in tool materials.
- With appropriately selected cutting parameters, turning with CBN and CC inserts can achieve low *Sa* values (0.4–0.6 µm), allowing the grinding operation to be omitted. Surfaces machined with CBN inserts were characterized by a more regular distribution of peaks and valleys, whereas CC inserts produced a wavy surface, indicative of intense plastic deformation of the surface layer. Additionally, low *Sa* values may be accompanied by single peaks, indicating that this parameter does not always fully reflect the presence of extreme surface irregularities.
- Local defects were observed on the surfaces machined with both CBN and CC inserts, including adhesive bonds of chips and tool material. For CBN inserts, micro-scratches caused by chip friction were also observed, while turning with CC inserts resulted in a side flow phenomenon associated with intense plastic deformation of the surface layer.
- It was confirmed that the use of CBN inserts in finish turning of hardened SSs is effective and comparable to the results obtained using commonly applied CC inserts with a PVD coating.

## Figures and Tables

**Table 1:** Coordinates of test points.

Point No.	1	2	3	4	5	6	7
*v_c_*, m/min	200	150	250	275	175	225	125
*f*, mm/rev	0.15	0.2	0.1	0.175	0.075	0.125	0.225

## Data Availability

The raw data supporting the conclusions of this article will be made available by the authors on request.

## Abbreviations

The following abbreviations are used in this manuscript:

BuE	Build-up Edge
CBN	Cubic Boron Nitride
CC	Cemented Carbide
CT	Conventional Turning
PCBN	Polycrystalline Cubic Boron Nitride
PCD	Polycrystalline Diamond
PSI	Parameter Space Investigation
SCD	Single-Crystal Diamond
SiC	Silicon Carbide
SR	Surface Roughness
SS	Stainless Steel
ST	Surface Topography
UVT	Ultrasonic Vibration Turning
